# Personal End-of-Life Healthcare Preferences Among Bereaved Surrogates to People With Dementia: A Vignette Study

**DOI:** 10.1093/geroni/igaf122.1320

**Published:** 2025-12-31

**Authors:** Rachel Bloom, Karen Siedlecki

**Affiliations:** Yale University, New York, New York, United States; Fordham University, New York, New York, United States

## Abstract

As the number of people living with dementia in the U.S. grows, more people are taking on the role of surrogate medical decision-making. Bereaved dementia surrogates have been uniquely exposed to end-of-life decision-making, observing what can go right and wrong and forming judgments around quality-of-life. This project utilizes a lifespan developmental perspective to gain greater insight into bereaved dementia surrogates’ preferences and attitudes towards end-of-life healthcare. Using vignettes, this study compared the end-of-life preferences of bereaved dementia surrogates (n = 108), bereaved non-dementia surrogates (n = 187), and non-surrogate controls (n = 697). Significant differences between the three groups were found in responses to the second vignette on memory loss and feeding, χ2(8, n = 992) = 33.88, p < .001, the third vignette on aspiration pneumonia, χ2(8, n = 992) = 46.15, p < .001, and the fourth vignette on feeding tube placement, χ2(8, n = 992) = 42.50, p < .001. Both surrogate groups were less likely to select the most aggressive treatment option in the second, third, and fourth vignettes. Controls were more likely than all surrogates to opt for aggressive treatment and less likely to opt for comfort care in the third vignette. Dementia surrogates were more likely to indicate they want measures to help them die peacefully in the fourth vignette, and less likely to respond “I don’t know.” These findings demonstrate how serving as a dementia surrogate can impact personal end-of-life healthcare preferences, with public health implications for an aging population.

